# Is there an association between mastitis and breast cancer? a retrospective cohort study from Germany

**DOI:** 10.1007/s10552-024-01909-w

**Published:** 2024-08-29

**Authors:** Vedanth D. Krishnan, Karel Kostev, Matthias Kalder

**Affiliations:** 1 Department of Gynecology and Obstetrics, University Hospital Marburg, Philipps-University, Marburg, Germany; 2 IQVIA, Frankfurt, Germany

**Keywords:** Mastitis, Breast cancer, Association, Gynecology, Germany

## Abstract

**Purpose:**

The aim of the study was to explore the association between mastitis and subsequent breast cancer.

**Methods:**

This retrospective cohort study included women aged ≥ 18 years with an initial mastitis diagnosis from 315 office-based gynecologists in Germany between January 2005 and December 2021. Women without mastitis were matched to women with mastitis using propensity score matching based on age, index year, average yearly consultation frequency during the follow-up period, and coexisting diseases such as obesity, benign mammary dysplasia, hypertrophy of the breast, unspecified lump of breast, and other disorders of the breast.

The 10-year cumulative incidence of breast cancer for the mastitis-cohort and non-mastitis-cohort was studied with Kaplan–Meier curves using the log-rank test. The association between mastitis and breast cancer was studied separately for four age groups with univariable Cox regression analyses.

**Results:**

In the follow-up period of 7 months to 10 years after the index date, 2.9% of mastitis patients and 2.4% of matched non-mastitis patients were diagnosed with breast cancer. A Cox regression analysis revealed a significant association between mastitis and subsequent breast cancer (HR: 1.37; 95% CI: 1.11–1.70). According to the age-stratified analyses, a strong and significant association was only observed in the age group > 50 years (HR: 1.73; 95% 1.25–2.40).

**Conclusion:**

The findings of our retrospective cohort study support an association between mastitis and subsequent breast cancer diagnoses in women aged > 50 years. The pathophysiological basis and possibility of confounders however requires further investigation.

## Introduction

Breast cancer is the most common malignant neoplasm in women around the world [1]. According to global cancer statistics [2], female breast cancer is overall the most commonly diagnosed cancer with 11.7%, and the fifth highest leading cause of cancer deaths with 6.9%, followed by lung (18%), colorectal (9.4%), prostate (8.3%), and stomach cancers (7.7%). In a paper from 2017, Sun and colleagues [3] reviewed the risk factors and preventions of breast cancer. Even though the pathogenesis of breast cancer has not been fully unraveled, risk factors for breast tumorigenesis include breast cancer stem cells (CSCs), tumor microenvironments, genetic as well as epigenetic environmental factors. Furthermore, tumor microenvironments can be mutagenic inflammatory generated by macrophages or neoplasms caused by stromal influences such as carcinogens.

Mastitis is defined as an inflammation of breast tissue, which is mainly caused through milk stasis and infection. Breastfeeding mothers can develop milk stasis due to incomplete removal of milk from the breast duct which in turn can cause blockage of the ducts. This is referred to as puerperal mastitis or lactational mastitis, which is more common than non-puerperal mastitis. Furthermore, in a review article conducted by Angelopoulou et al. [4], the mastitis-causing bacteria *Staphylococcus aureus*, *Staphylococcus epidermidis* and species of *Corynebacterium* have been identified as predominant etiological agents. According to estimates of the World Health Organization, the incidence of lactational mastitis varies from a few to 33% of lactating women [5]. Sore nipples pose a risk factor for mastitis and can be caused by infant attachment difficulties during breastfeeding. Infant mouth anomalies, such as cleft palate, bacterial infections or yeast infections can further contribute to sore nipples and subsequently mastitis [6].

In recent years, various studies on the association of mastitis with an increased risk of developing breast cancer have emerged. In 2020, Chen and colleagues [7] performed a retrospective population-based cohort study on Taiwanese women more than 40 years of age to determine the correlation between mastitis and breast cancer. They concluded that women with mastitis have an increased risk of developing breast cancer compared to the non-mastitis control groups. Based on previous significant population-based cohort studies, Nolan et al. [8] presented the hypothesis that mastitis is proposed to increase the risk of breast cancer. In addition, the authors discussed that the risk of breast cancer through mastitis, especially puerperal mastitis, can be mitigated through preventive measures that reduce the likelihood of developing mastitis. In a prospective population-based cohort study from 2002, Peters and colleagues [9] found a 37-fold increased risk of breast cancer diagnosis within 12 months of treatment for women with non-puerperal mastitis. However, the authors state that the incidence rate of breast cancer in the study is possibly overestimated due to a methodological bias. Furthermore, Chang et al. [10] concluded that the risk of breast cancer in women with non-puerperal mastitis is significantly higher than those without non-puerperal mastitis, especially for women aged less than 50 years, with a lower socioeconomic background and with hormonal medication. On the other hand, Lambe and colleagues [11] performed a retrospective population-based cohort study in Sweden by examining the possible associations between predominantly puerperal mastitis in patients and the subsequent risk of development of breast cancer. They were able to discover a slightly increased risk. However, they had investigated only hospitalized women, thus focusing on a much narrower cohort than the general population. A most recent study by Chang et al. [12] suggests that the transcriptional profile of inflammatory breast tissue was similar to ER-negative malignant tumors, hence associating inflammatory lesions with the development of breast cancer.

The aforementioned studies link the occurrence of mastitis with the development of breast cancer. However, they are often limited to certain age groups of women and focus on specific kinds of mastitis or inflammatory lesions rather than making a statement on mastitis in general.

Therefore, the aim of this retrospective cohort study is to investigate the association of puerperal mastitis as well as non-puerperal mastitis and the risk of breast cancer among patients treated in general practices in Germany. Furthermore, this study explores the hypothesis that women with a history of mastitis have an increased risk of developing breast cancer than those without mastitis. Published resources on the correlation between mastitis and breast cancer available to us seem very meager and limited. With the present retrospective extended cohort study, we propose to contribute to the volume of literature on the subject.

## Methods

### Database

This retrospective cohort study used data from the IQVIA ™ Disease Analyzer database that contains anonymous electronic medical records including baseline demographic data, diagnoses, and prescriptions from computer systems used in the office-based practices (Rathmann et al. 2018) [13]. This data source has often been used in previous studies focusing on breast cancer [14–16]. Rathmann et al. could show that the panel of practices included in this database is representative of general and specialized practices in Germany.

### Study population

**Fig. 1:**
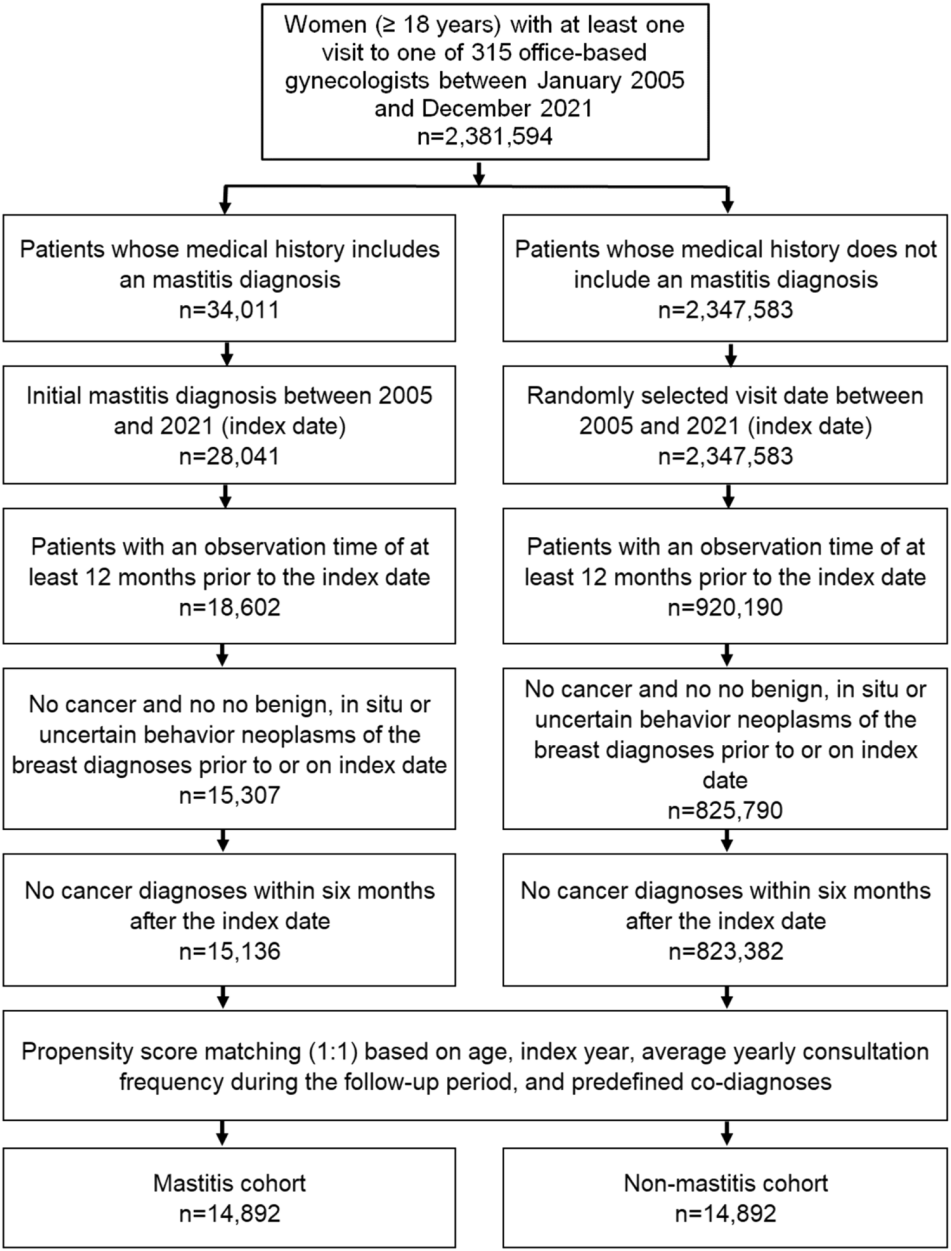
Selection of study patients

This study included women aged ≥ 18 years with an initial mastitis diagnosis (ICD-10: N61) from 315 office-based gynecologists in Germany between January 2005 and December 2021 (index date; Fig. 1). Patients were only included when they had an observation time of at least 12 months prior to the index date and no cancer diagnoses (ICD-10: C00-C97) and no benign, in situ or uncertain behavior neoplasms of the breast (ICD-10: D05, D24, D48.6, D49.3) prior to or at index date, or within six months after the index date. After applying similar inclusion criteria, women without mastitis were matched with women with mastitis using nearest neighbor propensity score matching (1:1) based on age, index year, average yearly consultation frequency during the follow-up, and pre-defined co-diagnoses documented within 12 months prior to or at index date (obesity (ICD-10: E66), benign mammary dysplasia (ICD-10: N60), hypertrophy of breast (ICD-10: N62), unspecified lump of breast (ICD-10: N63), and other disorders of breast (ICD-10: N64). For the non-mastitis cohort, the index date was that of a randomly selected visit between January 2005 and December 2021 (Fig. 1).

### Study outcomes and statistical analyses

The outcomes of the study were the initial diagnoses of breast cancer (ICD-10: C50) in the time seven months to ten years following the index date as function of mastitis. Differences in the sample characteristics and diagnosis prevalence between cohorts with versus without mastitis were compared using the Wilcoxon signed-rank test for continuous variables, and the McNemar and the Stuart-Maxwell test for categorical.

The 10-year cumulative incidence of breast cancer in the cohort with and without mastitis was studied with Kaplan–Meier curves using the log-rank test. Univariable Cox regression analyses were conducted to assess the association between mastitis and breast cancer. Cox regression analyses were conducted separately for two age groups (≤ 50 and > 50 years). As sensitivity analyses, regression analyses were repeated with elimination of breast cancer cases found within one year and two years after index date. Due to large patient samples, a p-value of < 0.01 was considered to be statistically significant. Analyses were carried out using SAS version 9.4 (SAS Institute, Cary, USA).

## Results

### Basic characteristics of the study sample

The present study included 14,892 women with mastitis and 14,892 women without mastitis. The basic characteristics of study patients are displayed in Table 1. Mean age was 39.2 (standard deviation (SD): 13.0) years. Patients visited their gynecologist in average 2.1 times per year during the follow-up. Due to matched pairs design, no significant differences were observable between two cohorts in terms of age, visit frequency, and co-morbidities (Table 1).

**Table 1:** Baseline characteristics of the study sample (after propensity score matching)

Variable Mean (± SD)	Proportion among women with mastitis (N = 14,892)	Proportion among women without mastitis (%) (N = 14,892)	p-value
Age	39.2 (13.0)	39.2 (13.0)	1.000
Age ≤ 30	4040 (27.1)	4040 (27.1)	1.000
Age 31–40	5622 (37.7)	5622 (37.7)	
Age 41–50	2453 (16.5)	2453 (16.5)	
Age > 50	2777 (18.7)	2777 (18.7)	
Number of physician visits per year during the follow-up	2.1 (1.5)	2.1 (1.5)	0.500
Index year			
2005–2008	1832 (13.5)	1832 (13.5)	
2009–2012	2675 (19.7)	2675 (19.7)	1.000
2013–2017	5272 (38.9)	5272 (38.9)	
2018–2021	3791 (27.9)	3791 (27.9)	
Diagnoses documented within 12 months prior to or at index date			
Obesity	1397 (9.4)	1349 (9.1)	0.336
Benign mammary dysplasia	1280 (8.6)	1280 (8.6)	1.000
Hypertrophy of breast	247 (1.7)	230 (1.5)	0.433
Unspecified lump of breast	289 (1.9)	254 (1.7)	0.130
Other disorders of breast	4485 (30.1)	4337 (29.1)	0.060

Proportions of patients in N and % given, unless otherwise indicated. SD: standard deviation.

### Association of mastitis with subsequent breast *cancer*

**Fig. 2:**
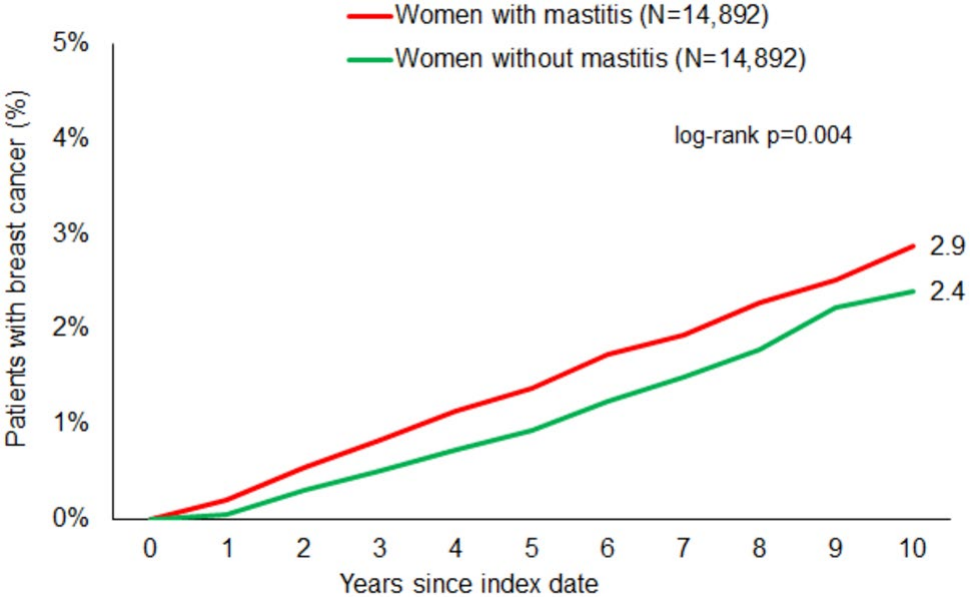
Cumulative incidence of breast cancer in individuals with and without mastitis

In the time 7 months to 10 years after index date, 2.9% of mastitis patients and 2.4% of matched non-mastitis cohort (p = 0.004) were diagnosed with breast cancer (Fig. 2).

In the regression analysis, there was a significant association between mastitis and subsequent breast cancer (HR: 1.37; 95% CI: 1.11–1.70) (Table 2). However, in age-stratified analyses, no significant associations were observed in age group ≤ 50 years although the incidence rates were higher in mastitis cohort in each age group. In the age group > 50 years, a strong and significant association between mastitis and breast cancer was observed (HR: 1.73; 95% CI: 1.25–2.40) (Table 2). The positive association between mastitis and breast cancer was confirmed in sensitivity analysis with elimination of breast cancer cases found within one year after index date (HR:1.63; 95% CI: 1.16–2.32), but not when breast cancer cases within two years after index date were eliminated (HR: 1.39; 95% CI: 0.94–2.05) (Table 2).

**Table 2:** Association between mastitis and subsequent breast cancer in women followed by office-based gynecologists in Germany (univariable Cox regression models)

Age group	Breast cancer cases and follow-up time in years in women with mastitis	Incidence (cases per 1000 patients years) in women with mastitis	Breast cancer cases and follow-up time in years in women without mastitis	Incidence (cases per 1000 patients years) in women without mastitis	HR (95% CI)	P value
*Breast cancer in the time 7 months to 10 years after index date (n = 14,892 matched pairs)*						
Total	213 / 71,091	3.0	138 / 63,661	2.2	1.37 (1.11–1.70)	0.004
Age ≤ 50	116 / 59,907	1.9	27 / 11,944	1.5	1.23 (0.93–1.64)	0.147
Age > 50	97 / 11,084	8.7	57 / 11,374	5.0	1.73 (1.25–2.40)	0.001
*Breast cancer in the time 13 months to 10 years after index date (n = 14,857 matched pairs)*						
Total	188 / 71,065	2.6	128 / 63,414	2.0	1.29 (1.03–1.61)	0.027
Age ≤ 50	105 / 59,895	1.8	77 / 52,244	1.5	1.16 (0.87–1.56)	0.312
Age > 50	83 / 11,170	7.4	51 / 11,170	4.6	1.64 (1.16–2.32)	0.006
*Breast cancer in the time 24 months to 10 years after index date (n = 14,780 matched pairs)*						
Total	150 / 70,945	2.1	105 / 63,366	1.7	1.24 (0.96–1.59)	0.096
Age ≤ 50	90 / 59,832	1.5	62 / 52,178	1.2	1.23 (0.89–1.69)	0.218
Age > 50	60 / 11,113	5.4	43 / 11,188	3.8	1.39 (0.94–2.05)	0.102

## Discussion

Our study revealed a significant association between mastitis and subsequent breast cancer in the time seven months to ten years and 13 months to ten years after index date in women over 50 years of age. However, no significant association was observed when breast cancer cases within the first two years after index date were eliminated. Similar observations were made by Chen and colleagues., who also concluded a greater risk of breast cancer events in Taiwanese patients with mastitis aged above 50 years with an HR of 5.46 (2.31–12.87). Another population-based cohort study presented by Chang et al., focusing specifically on non-puerperal mastitis patients, demonstrated a significantly higher risk of breast cancer in women with non-puerperal mastitis compared to the control group of women without mastitis (aHR = 1.94, 95% CI: 1.30–2.90). Likewise, women aged > 40 years showed even higher risk of breast cancer, which are in par with our own observations. Finally, another cohort study conducted by Lambe and colleagues found an increased incidence rate of breast cancer in women who have been hospitalized with mastitis compared to women without mastitis with an IRR of 1.22 (1.01–1.48).

The age-stratified analyses in our study reveal that the cumulative incidence of breast cancer diagnoses in mastitis patients rise with increasing age, and furthermore show a strong and significant association in women aged above 50 years. This association can be seen across multiple studies in the past. Indeed, aging itself and reproductive factors such as early menarche or late menopause can increase the risk of breast cancer, as shown in the study by Sun and colleagues [3]. Correspondingly, an early age at first pregnancy and higher number of childbirths are seen as protective factors against the development of breast cancer. On the one hand, parity therefore decreases the risk of certain types of breast cancer [17]. On the other hand, every childbirth carries the risk of developing puerperal mastitis, which can consequently be associated with an increased risk of breast cancer, as demonstrated in this study.

According to Nolan and colleagues, bacterial infection following puerperal mastitis stimulates the development of a premalignant environment, which in turn increases the risk of breast cancer. There are common characteristics between oncogenesis and bacterial infection through *Staphylococcus aureus* for instance hypoxia and environmental acidification to extracellular pH levels between 6 and 7 [18].

There are several studies that positively associate mastitis to breast cancer in addition to the aforementioned correlations. As early as the 1970s, studies discovered that patients with chronic mastitis or chronic cystic mastitis develop breast cancer more frequently than women without any form of mastitis. Specifically, Monson and colleagues [19] found that breast cancer development was 2.5 times more likely in their cohort study, whereas Donelly et al. [20] observed a 2.9-fold increase.

Furthermore, recent reports highlight the occurrence of breast cancer either as a coexisting entity or as a result of granulomatous mastitis. In February 2024, Salih and colleagues [21] presented a case report, in which the initial diagnosis of recurring granulomatous mastitis proved to be masking an underlying ductal carcinoma in situ (DCIS). Similarly, in a case presented by Lugman et al. [22], chronic granulomatous mastitis was found to be a possible precursor for malignancy of the breast. These publications illustrate the importance of the search for a second pathology when dealing with granulomatous mastitis. Other studies found an association between granulomatous lobular mastitis and ductal carcinoma. A distinction is made between an invasive ductal carcinoma and ductal carcinoma in situ, however DCIS can progress to invasive cancer if left untreated. Limaiem and colleagues [23] presented a case of granulomatous lobular mastitis coexisting with an infiltrating ductal carcinoma. However, the question whether breast cancer arises from granulomatous lobular mastitis was put on hold, since the literature was scarce and inconclusive. Since then, Oddó et al. [24] and Evans et al. [25] among other groups have contributed findings that link ductal carcinoma to granulomatous lobular mastitis.

The risk of developing breast cancer is increased by inflammatory diseases, as shown by Bhatelia and colleagues [26]. More accurately, it shows that tissue like receptors (TLRs) are key players in activating inflammatory pathways and creating favorable tumor microenvironment. Regarding different inflammatory diseases of the breast, a distinction is made between mastitis as inflammation of the breast tissue and inflammation of tissues within the breast, caused by other sources. Exemplary are obesity-associated inflammations, which correlate with breast cancer, as shown by various studies [27–30].

### Strengths and limitations

This retrospective cohort study has several strengths. First, the large dataset used represents collected data from 315 office-based gynecologists in Germany. Second is the long duration of 10 years of follow-up after initial diagnosis. Finally, this study used narrow inclusion criteria that incorporated 12 months observation time prior to index date and propensity score matching based on age, index year, consultation frequency, and co-diagnoses to ensure comparability between the cohort groups. However, our study is subject to methodological limitations. For instance, there may be irregularities in the treatment of mastitis patients and non-mastitis patients. It is possible that doctors examined the breast of mastitis patients more closely or accurately compared to those in the corresponding cohort group. This can lead to an earlier detection of breast cancer or discoveries of cancer that otherwise would not have been found. Furthermore, it can result in false-positive findings. It is important to note possible observational errors during the diagnosis of mastitis, as it could in fact be undetected breast cancer tissue. Even if these cases do not occur frequently, it is advisable to carry out a detailed examination for breast cancer in cases of mastitis. This study does not take into account, which examinations were performed during the follow-up consultations in the gynecological practices. Breast lumps due to mastitis could be an incentive for doctors to focus inspections on the breast region and therefore result in a performance bias. Lastly, despite the sizable amount of patients this study lacks information regarding specified diagnoses, patient histories, and risk factors as well as hospitalization and mortality details. There is no distinction made between lactational, non-lactational mastitis, acute or chronic mastitis subtypes nor their severities. We were not able to control for reproductive factors such as parity, age at first birth, and breastfeeding practice and duration. As these factors are clearly associated with both breast cancer incidence and mastitis, they therefore could be potent confounders. Moreover, detailed patient information on smoking habits, alcohol-consumption, sexual behavior, obesity, family history as well as prior medical histories would have allowed for a more comprehensive assessment on the risk of breast cancer. Therefore, various factors that may have affected the cumulative incidence of breast cancer have not been taken into account.

In summary, we found an association between history of mastitis and development of breast cancer in women aged > 50. Statistical significance is particularly noted in breast cancer diagnoses that occurred in closer proximity to initial mastitis diagnosis, and we cannot entirely rule out that the observed associations may be explained by breast cancers being initially diagnosed as mastitis.

Therefore, this association should be explored further in prospective studies that are better able to control for confounders.

## Data Availability

The datasets used and analyzed during the current study are available from the corresponding author on reasonable request.
